# The implementation evaluation of primary care groups of practice: a focus on organizational identity

**DOI:** 10.1186/1471-2296-11-15

**Published:** 2010-02-22

**Authors:** Charo Rodríguez, Marlei Pozzebon

**Affiliations:** 1 Area of Health Services and Policy Research, Department of Family Medicine, McGill University, Montreal, Québec, Canada; 2 Current Address: Department of International Business, HEC Montréal, Montréal, Québec, Canada

## Abstract

**Background:**

Since 2002 the Health Ministry of Québec (Canada) has been implementing a primary care organizational innovation called 'family medicine groups'. This is occurring in a political context in which the reorganization of primary care is considered necessary to improve health care system performance. More specifically, the purpose of this reform has been to overcome systemic deficiencies in terms of accessibility and continuity of care. This paper examines the first years of implementation of the family medicine group program, with a focus on the emergence of the organizational identity of one of the pilot groups located in the urban area of Montreal.

**Methods:**

An in-depth longitudinal case study was conducted over two and a half years. Face to face individual interviews with key informants from the family medicine group under study were conducted over the research period considered. Data was gathered throuhg observations and documentary analysis. The data was analyzed using temporal bracketing and Fairclough's three-dimensional critical discourse analytical techniques.

**Results:**

Three different phases were identified over the period under study. During the first phase, which corresponded to the official start-up of the family medicine group program, new resources and staff were only available at the end of the period, and no changes occurred in medical practices. Power struggles between physicians and nurses characterized the second phase, resulting in a very difficult integration of advanced nurse practitioners into the group. Indeed, the last phase was portrayed by initial collaborative practices associated with a sensegiving process prompted by a new family medicine group director.

**Conclusions:**

The creation of a primary care team is a very challenging process that goes beyond the normative policy definitions of who is on the team or what the team has to do. To fulfil expectations of quality improvement through team-based care, health care professionals who are required to work together need shared time/space contexts to communicate; to overcome interprofessional and interpersonal conflicts; and to make sense of and define who they collectively are and what they do as a clinical team.

## Background

The general purpose of this research is to assess the implementation of family medicine groups in Quebec from the point of view of their organizational identity. As in the case in many other Western countries, the Canadian primary care system has endured several reforms over the last several years [1]. Health care organization and management in Canada falling under provincial jurisdiction, in 2000 the Quebec Government instituted the 'Commission for the Study of Health and Social Services' (known as the 'Clair Commission') [2] to assess the state of the provincial health care system and to propose alternatives to better face its current challenges. In the opening pages of its report, the Clair Commission indicates the existence of important organizational problems, mainly in terms of accessibility and continuity of care. To overcome these systemic deficiencies, the Commission made a number of recommendations and propositions. Those lately retained by the Quebec Health Ministry concerned family physicians and involved the implementation of a primary medical practice innovation called 'family medicine groups' (FMGs).

In the publicly funded Quebec health care system, the vast majority of primary medical care has been traditionally provided by practitioners working in private clinics or polyclinics and who are remunerated on a fee-for-service basis. Relatively few physicians work in community care centres (*centres locaux de services communautaires *or CLSCs) (i.e. equivalent to 12-14% full-time physicians) [3,4] or in family medicine units, the latter being located both in hospital and CLSC settings. In addition, family physicians display a very heterogeneous practice profile, the majority dividing their time between two or more organizations. Furthermore, a particular characteristic of the Quebec health care system is that about 40% of all family physicians work at the secondary level of care [3,4].

Heightening interprofessional co-operation as well as inter-organizational collaboration, the Clair Commission drew up the following general features for the Quebec FMG: (1) a group practice of 6-10 family physicians, (2) working in close collaboration with 2-3 advanced nurse practitioners, (3) delivering 24-hour front-line services to a rostered clientele, (4) networking with specialized and ultra-specialized health services, and (5) being paid according to a mixed scheme (i.e., capitation, fee-for-services and lump sum). This set of characteristics constitutes an initial standard view of a FMG that necessarily "confronts" local contingencies when put into operation. In this sense, it is interesting to note that physicians and nurses, even if they create a new group-based practice, continue to work in their usual primary care facilities. Additionally, in most cases these professionals share these 'old' organizational spaces with people not involved in the FMG pilot project - for instance, the 20 family physicians that constitute the FMG under study make up part of a pool of 44 operating in the same facility. In other words, a complex confluence of 'old' and 'new' primary care organizational forms is taking place. In addition, "advanced nurse practitioner" is not yet a legal profession in Quebec and, consequently, the current reform of front-line services in the province is contingent on important changes in the profiles of nursing practice. Furthermore, interprofessional (e.g., family physician/nurse) and inter-organizational relations between different levels of care (e.g., private clinic/CLSC, private clinic/hospital), particularly regarding medical care, are not yet well established [5-7]. In this context of cohabitation of old and new organizational spaces, a lack of tradition of networking medical care and intense political attention from provincial and federal levels concerning front-line services, one may expect that the construction of FMG identity would be an extremely complex, multifaceted, and conflicting process. This would be particularly challenging in urban contexts, where the number of actors involved is significantly greater than in peripheral, rural and remote areas. Thus, our research question has been stated as follows: (1) what is the meaning of an urban FMG identity and how does it emerge and become legitimated in this particular political context?

Existing evidence suggests that organizational success appears to be positively linked with organizational identity [8,9]. Accordingly, we argue that, in the field, the success of the FMGs program and the role that these new groups of practice will play in the Quebec health care landscape will be intimately linked with FMGs identity [10]; in other words with how FMGs members *think*, *talk*, and ultimately *behave *as FMGs.

Identity is a complex topic that has been treated differently in organizational literature. Some traditions tend to consider organizational identity as a set of essential and rather static elements that characterize organizations [11]. In contrast, other perspectives challenge this position and consider that identity is a socially and historically constructed dimension that is constantly subject to contradictions, revisions, and change [12]. As noted by Oliver and Roos [13], organizational identity is viewed as "a product of intersubjective, shared perceptions and views of 'who' an organization is."

Shaped thus, the aim of the present study is to understand the social construction of the identity of a FMG. We are particularly interested in understanding the manner in which actors use their *power *in the social activity of the identity construction of a family medicine group. Identity is thus as a complex and dynamic organizational construct, being constantly shaped and reshaped by organizational actors through an ongoing *political *process [14]. Furthermore, we see actors' *discourses *as constitutive of both their own identity and the space and role they occupy in the social world [15].

## Methods

This is an in-depth longitudinal qualitative case study [16] and encompasses the period from October 2002 to April 2005, for which we obtained appropriate ethical approval by the McGill University Faculty of Medicine Institutional Review Board (Reference A04-E08-03A). Using Stake's typology, our case study is both intrinsic and instrumental: intrinsic because the case displays an interest in itself as a pilot group and instrumental because it is also examined in order to advance knowledge about the particular topic under scrutiny, i.e. the social construction of an urban FMG identity. Hence, the case selected shows some similarity to other urban FMGs and seems to offer to us a good "opportunity to learn" [16].

It is a FMG located in Montreal and was one of three initial pilot FMGs located in the Montreal Metropolitan Area. It comprises 20 family physicians (equivalent to 10 full-time) from a private clinic and a university family unit created in a community hospital setting, two nurses considered staff of the neighbourhood community health centre, and two administrative staff members. The private clinic is located just opposite the hospital and the community health centre is not far away. The population living in the neighbourhood is economically under-privileged and is rapidly aging. In addition, the territory is relatively poor in medical resources.

Adopting what has been called a 'conflict and bargaining' perspective for program evaluation [17,18], we have combined two theoretical and methodological approaches, namely structuration theory and critical discourse analysis [19]. On the one hand, and in agreement with authors such as Sarason [20] and Brocklehurst [21], we consider that the recursive relationship between agency and structure proposed by Giddens' structuration theory [22] constitutes a powerful framework for better understanding the social construction of organizational identity. Structuration theory challenges the functionalist notion of social structure as something external to human agents, constraining their actions from outside. Instead, structure for Giddens consists of recursively organized sets of rules and resources that, acting as memory traces, are instantiated in human actions: "The structural properties of social systems exist only insofar as forms of social conduct are reproduced chronically across time and space" [22]. Furthermore, in virtue of the duality of structure, structure is viewed as both the medium and the outcome of the social conduct it recursively sets up. In other words, human actors' agency is influenced by the structure of the social systems in which they live and at the same time these knowledgeable human actors may, through their conduct, modify the structural properties of such systems. This is the notion of social structure adopted in this study on identity.

On the other hand, we think that discursive organizational practices play a crucial role in the process of identity construction [23,24]. Organizational discourse "refers to the structured collection of texts embodied in the practices of talking and writing (as well as a wide variety of visual representations and cultural artefacts) that bring organizationally related objects into being as these texts are produced, disseminated and consumed" [25]. Among the great variety of discourse analysis traditions, we are concerned with critical discourse analysis, which is considered one of the most context-sensitive discursive perspectives [25,26].

We want to point out that a constructivist ontology that assumes that organizational (social) phenomena are constantly socially produced, largely discursively, does not imply that social life is exclusively constructed in discursive activity: non-discursive activity also matters. As Giddens notes, discursive activity is a key part of what people do, but not everything they do, structure being expressed in the things people discursively and non-discursively do "in a regularized and institutional way" [27]. Having said this, we also consider that some organizational phenomena are of a greater discursive nature than others. We locate identity among the former, an assumption that fully justifies our emphasis on organizational discourse activity in the examination of the structuration of identity.

In terms of research methods, we first used face-to-face individual semi-structured interviews with different members of the FMG. These participants were members of the group who could provide the richest information about the process of the social construction of the FMG, i.e. key physicians, nurses, and administrative staff (purposeful sample). They were identified following a snowball sampling strategy. Although informal contact with the director of the FMG began in October 2002 (date of the official creation of the group), the fieldwork began on April 2003, when ethical approval was obtained. A total of 11 interviews were thus carried out between spring 2003 and spring 2005. They lasted 50 to 140 minutes and were conducted by the first author. Before each interview, participants were invited to carefully read and sign a consent form, which had previously been approved by the university ethics board.

Interviews were complemented by the first author's participant observation of all the meetings held by the FMGs Montreal Regional Board as of October 2003 (a total of 10 meetings in this forum of representatives of all the FMGs in the region) and of a number of the FMG administrative meetings. Researcher's participation in these meetings was always allowed, but the degree of participation varied according to the type of meeting and the content of discussions.

A variety of documents were gathered during the period considered (October 2002 to April 2005), such as proceedings and minutes, formal agreements, media articles and government reports.

**Figure 1:**
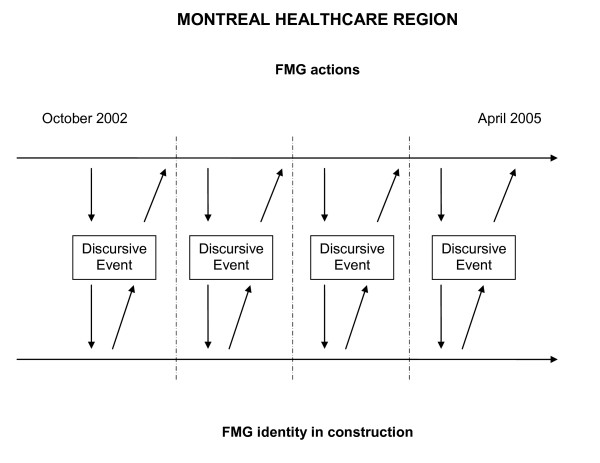
**Combining temporal bracketing and critical discourse analysis strategies**.

We have combined temporal bracketing strategy [28] with Fairclough's framework [29] (see Figure 1). Following these strategies, we have first broken down the whole period considered into sub-periods. Although we have gone back and forth over the texts during the analysis, temporal bracketing constitutes the first analytical step performed due to our intense presence in the field; this has allowed us to identify critical FMG moments of change as "discontinuities" at the transitions to the different sub-phases, for instance, the hiring of nurses or the appointment of a new FMG director. Then, in each sub-period, we have looked for pieces of text from interviews and minutes of the FMG meetings that highlight power struggles (e.g. between physicians and nurses), the defence of particular actors' interests, or important decision-making processes. As for discursive practice, our focus has been on the interpretation of metaphors. Metaphors are those figures of speech in which a word or a phrase that literally denotes an idea or object is used in place of another, suggesting analogy between them. Metaphors help understand organizational change, and are powerful tools through which "future identities are made" [30].

Then, based on the piece of text being analyzed, but also considering other texts (intertextual analysis) from the local (fieldwork notes), regional (minutes from the FMGs Montreal Regional Board meetings), and provincial health care context (newspapers articles, governmental reports), we have provided plausible explanations of the progressive structuration of the FMG identity. Indeed, explanations have been integrated into a coherent narrative, first by sub-period and then in a more general one corresponding to the whole research period considered.

We stress that, although an initial data analysis was performed by the first author, both authors were involved in final stages of analysis and interpretation. Furthermore, in order to strengthen the credibility of the study, an initial research report was sent to the participants for their feedback.

## Results

Over the two-and-a-half year period included in this paper, we have identified three interconnected phases.

### First phase: (October 2002 - October 2003) - Driven by the ideal model of primary health care delivery

The first phase of our analysis encompasses the first year of FMG implementation, from October 2002 to October 2003. Administrative staff was hired in the summer of 2003 and nursing staff was integrated into the group in October 2003. This period was characterized by vague governmental directives regarding the project of reform. At the local level, the process was distinguished by its slow pace, the FMG first "existing" through the idealistic enthusiasm of its champions.

From October 2002 to March 2003, the prevailing idea of the FMG was that if a number of physicians decided to work together in a group practice, they could better facilitate the case management of their clientele. However, the need for creating such a group of practice was exclusively sustained by the two physicians who championed the project in this FMG, one the director of the family medicine unit located at the hospital and the other a counsellor in public health and a clinician working in both the hospital and the private clinic. The remaining physicians, very busy professionals, were not really motivated to embark on the project. Physicians are very powerful actors who can easily be resistant to changing practice modes if they do not see a clear and immediate advantage to doing so. Strategies to be adopted in order to support a transition from sole to group practice should therefore be as "unnoticeable" as possible, as is explained with the metaphor of "the engine bathing in oil":

As my secretary says, there is the need to settle the everyday problems; they [the physicians] are overwhelmed with clinics, so the last thing they want is to be bothered by administrative issues. I have always said to them that I will try to build such an effective FMG that they won't even realize its existence. OK! Things must run as if they are bathed in oil. Nobody hears an engine bathed in oil. We push on the accelerator, it runs, and then we slow down to stop. This is what I want.

The two initial champions of the project truly believed in the advantages of a medical group practice vis-à-vis the current state of primary health care delivery in Quebec, which is depicted with the metaphor of "the image on the water":

Over the last 20 years, I have seen deterioration in primary care services delivery towards sole practices. And each one is always convinced that he is offering the best possible service. Each one is giving the best of himself but, *grosso modo*, it is the image on the water. A group practice offers a better service to our patients. It is a question of re-establishing the model that was in place when I started to work in the 1980s, a more... ideal model, ideal; closer to the ideal of practice.

This enthusiasm of the initial champions prevailed throughout the initial period of this FMG's existence, despite the scepticism of the other physicians ("we respect what you are doing but we are not interested in it"), and also the difficulties present in the provincial context since the beginnings of the project. In this sense, the metaphors "dropping a bomb" and "by the skin of our teeth" show how the strong interest in the project by the initial champions went beyond the local context, making this FMG one of the first selected despite external constraints:

You know? Each Minister has his own personality. Mr. [T], people know him, it is his manner to act, he drops a bomb and then he initiates the change. So he announced that he wanted to implement new practices with family medicine groups, and then suddenly he dropped the bomb [the initial list of FMGs selected] when nobody in the Ministry even knew about it.

... [W]e wrote the application very quickly, and submitted it without even talking to the physicians here in order to know whether or not they agreed. So [F] and I sent the application, and it was refused. Later on we knew that another FMG would be selected. Then [F] re-wrote the application, [...] And then we were selected, but I think that we won by the skin of our teeth, mostly because we proposed to invest in a private clinic.

In March 2003, the FMG received an allocated budget of about CDN $300,000 to hire an administrative coordinator and a secretary and to cover operation expenditures. At the same time, a contract of services with the community health centre was established, patient enrolment began, and the search for two nurses was initiated. It was established that for each patient enrolled, his or her physician would receive a fixed annual amount of CDN $7, with a supplement of CDN $14 if the patient was considered vulnerable. Despite this financial incentive, very few physicians initiated the enrolment of their clientele. When hired in summer 2003, the administrative staff undertook this task.

Indeed, despite the new resources allocated, the enthusiasm of its champions and the "unnoticeable" strategies adopted in a general context of political vagueness were not enough to provoke structural change in medical practice in this FMG, which remained mainly an "idea" in the minds of its initiators. Nurses were finally hired in October 2003, constituting the transition to the second phase of the project.

### Second phase: (October 2003 - October 2004) - The difficult integration of nurses

This second phase, which covers the period from October 2003 to October 2004, in fact constitutes the first operational stage of this FMG pilot project. Not surprisingly, the difficulties of putting an ideal model of health care delivery based on interprofessional cooperation in place emerged rapidly. This period was mainly characterized by the difficult integration of two nurses into the FMG. In January 2004, they participated for the first time in a FMG meeting. At this meeting they highlighted that "an interprofessional collaboration asks for a shared vision of the specific professional practice of each member, and the development of relationships characterized by collegiality and trust, which will support client satisfaction" (minutes of the FMG group meeting on January 23, 2004). They also presented their vision of the role of a FMG nurse and the form for patient reference from physicians to nurses.

Reproducing traditional professional struggles in the health care sector, the difficult nature of the physician-nurse interprofessional relationship during this period was so intense in this group that the nurses would not attend the FMG meetings again until April 2005. Responding to the physicians' initial lack of interest in the nurses' work, as well as to a strong reaction to this from nurses, physicians and nurses met separately over this period, the latter also meeting very frequently with the administrative staff. The distance between physicians and nurses was explained by some physicians in the context of gender differences in the manner of delivering services, referring to "masculine/feminine poles":

For them [nurses], it is like they need feminine and masculine approaches. I see you frowning and she frowns. At home my wife reads the instructions before switching on the machine; but I try it, you know? [...] It is as two poles. They need to be in control before seeing patients; they need to be sure that they master different situations, to go to educational training, and well! But me, as an impatient doctor, I find this long.

In contrast, the nurses considered that physicians strongly resisted changing their practice modes ("How do you want me to put bread and butter on the table?") because, indeed, they did not want to share patient care responsibilities (i.e. power) with them. This was even the case with one of the physicians who more enthusiastically defended the "idea" of FMG in the preceding phase of the project:

When we tell them: "Yes, but there are clients who are not cared for, they are waiting to see a physician. Why do not you see the added value of telling them that they can go and see someone elsewhere? [...] Then they answer: "How do you want me to put bread and butter on the table?" This has been very difficult. Even Dr. [M] told us very recently - and this cut our breath - that he did not know whether he really wanted to change his practice! Because changing his practice means giving us more space, and then accepting new clients.

To explain their reactive position, one of the nurses also complained that, as a profession, nurses always have to take the first step toward settling interprofessional differences and opposed styles of health care delivery, an experience that she expressed with the metaphor "nurses as nuns":

[N]urses were also those who took everything on their shoulders. And I have always been a bit against the current. Because I think that when we adopt the role of supporting everything on our shoulders, this can act against us. We nurses want to do everything. We are still like nuns inside us at this level.

For the administrative staff, the FMG sense of self and its implications for medical clinical practice had also not yet developed. In this phase, these workers considered that the FMG "cornerstone" was still only constituted by nurses and administrative staff:

Us, our FMG team, we are the two nurses, and [administrative staff]. There is really a cornerstone here, but the point is to graft everyone to it. [...] Our FMG, it is really us. We have big discussions about what we would like to become, about who we are now, and what to do to become what we would like to. [...] But currently, everyone, the physicians, they have other worries. They are very busy. I think that they see what a FMG is, but they are not ready to stop and reflect on what they do... when they see a patient, and then to stop and reflect on "Should I send this patient to the nurse?" You know, they are not ready yet ...Because this implies a big questioning of their practice. I think they are not there yet.

In terms of new material resources, over this period the FMG physicians decided to accept the implementation of a computerized drug prescription system offered by the Health Ministry to all the FMG pilot projects.

At the same time, patient enrolment continued slowly, its rate progressing quicker for physicians working at the private clinic than for those working at the hospital family medicine unit. To explain the reason for this, the metaphor "to make the vase overflow" emerged: "Here [at the hospital family medicine unit] we have big problems. Big problems refer to [the idea that] that any additional task makes the vase overflow, a vase which is already full."

In summary, the first period of "official existence" of this FMG was characterized by a combination of an idealistic political discourse and prevailing values and norms of practice, which resulted in a FMG without a 'sense of self'. This lack of FMG identity led to a second phase, during which historical power games between physicians and nurses were reproduced in the daily activities of the clinical group.

### Third phase: (October 2004 - April 2005) - A new group leader who makes a difference

The third initial phase of the FMG pilot project started in October 2004, when a new director of the FMG, a female physician, was appointed at the request of the former director, who did not want to remain in his post and who appeared rather overwhelmed by the tasks required to make the FMG operational. In this sense, some administrative staff expressed, with the metaphor of "very good seller, little doer", how his abilities were profitable for the group in the first phase, but not when the moment to materialize the vision had come:

It is different. Dr. [M] was rather an intellectual. He thought, but acting... He was a very good seller, but a little doer. Dr [G], I think that with her, it is more complete. In addition to having a vision, all that stuff..., she has taken the contract and sees everything that must be done, to the letter. So the FMG is working, it is working more.

The new director also acquired great legitimacy vis-à-vis the nursing staff almost immediately. In the nurses' opinion, after a very difficult period, she was able "to keep the ship afloat", that is to bring a new and promising dynamic into the group that could support the FMG while it came into being:

We do not yet know whether or not physicians have a common vision of what a FMG is. This has not changed yet. However, what has changed is that there is a new person responsible for the FMG. And this makes a great difference. [...] Prior to this she obtained information about what a FMG is, about the role to be played by the director, about the issues concerning a FMG. She also obtained information about the other FMGs in Montreal. So when she accepted, she already knew what she was getting involved in. Also, I think she probably holds the qualities of a physician closer to the FMG vision. [...] So to keep the ship afloat, when it was sinking, and to do something. So after 6 months, it is not the same dynamic at all; it is not the same dynamic as before.

Concerning material resources, the acquisition of the computerized drug prescription system had not had a substantial impact on clinical practices yet because only one physician, who was very interested in informatics, used it regularly, but not without trouble. As was the case in other FMGs across the province, some of the problems often encountered were that the system got bogged down for several days, there were a multiplicity of passwords, the software was not adapted to clinical needs, too much time was devoted to use and training, and there were no link with pharmacists. As a result, only very few physicians working in FMGs "switch [ed] on their computers".

In terms of services, the official existence of this FMG did not have a significant impact on health delivery. The majority of physicians working in the private clinic kept practicing in a more or less sole fashion, and physicians working in the hospital family medicine unit maintained their usual clinical and medical training dynamic. So far, only services delivered by nurses were "new", although very few physicians referred patients to them, and those who did so were mostly residents training in the unit. However, for the new FMG leader, this was the great advantage of creating a FMG in a family medicine unit: "We are a family medicine unit and the big advantage for us is to work with a nurse; better, with two! Because if we look at other advantages there are not much more for physicians here." Thanks to the new director's emphasis on interprofessional co-operation, the nurses even agreed to "sell their business" to physicians in the FMG meeting held on April 10, 2005:

... [T]omorrow you will attend our meeting... So I have said to the nurses, the first 10 minutes, they will present what they are doing: "You will put all that on PowerPoint. That will be very interesting for physicians. Ten minutes. No political discourse, no mention of the legislation, etc. Just what you are doing, what kind of patients you are seeing. In doing so, you will make them realize what you are able to do. Then, they will start to be aware, and refer patients to you. But if you do not sell them your business, they will not know what you do and so they won't refer patients to you. At each meeting, you will come again, in order for physicians to become used to referring patients to you.

Another important point regarding the FMG identity and image was that not even its closest stakeholders knew what the FMG means and does, not patients ("I think they have no idea of what we are talking about") and not hospital clinical staff and managers. The lack of awareness of what a FMG is for the latter was justified through the metaphor "to have other fish to fry": "I sat down with administrative staff from the hospital, but they did not know what the FMG is doing at all. What we understand is that they have other fish to fry."

Indeed, during this third phase, a new female FMG director who believed that the main advantage for physicians to constitute a FMG was to work with nurses was appointed. Under her positive influence, discourses produced in this period brought back the conflictive interprofessional FMG dynamic that characterized the earlier phase, and began constructing a new one where, recognizing the challenging task of giving shape to the FMG, new local spaces for discussion towards co-operation emerged.

## Discussion

"At the end of 2002, the Premier Bernard Landry announced the creation of the first six family medicine groups (FMGs). Objectives were ambitious: a hundred FMGs should have been born over the year 2003 and their number should have been increased to three-hundred in 2005. However, only a hundred have been implemented this year and the concept is of little interest to family physicians that have not adhered yet. According to a recent poll carried out by the Quebec General Practitioners Federation, 71.3% of the latter have no intention of becoming a member of a FMG in the future."

(L'actualité médicale, March 16, 2005, 8)

This excerpt clearly illustrates how the Quebec FMG project has fallen short of its intent. By the time of writing these lines, about 178 FMGs have been accredited across the province, but only 18 of them (11.25%) operate in the Montreal urban area, 13 (65%) are located in private clinics, 5 (25%) in family medicine units and 2 (5%) in community health centres. In addition, only 11% of full-time equivalent family physicians are currently operating in these organizations [4]. This institutional context helps understand the difficult process of FMG identity construction in the period considered, particularly in urban areas, where the uptake of the FMG model by physicians has been much less intense than in other territories.

Considering the crucial role played by organizational identity in organizational life [10], our aim in this investigation was to examine the emergent identity of a new primary care group of practice during the first years of its official existence. Current imperatives of quality and cost-containment lead a current renewed interest in team-based care [31-33]. However, as Firth-Cozens notes: "Real teams do not just happen; they need to be developed both as groups and through their leaders..." [31]. Empirical evidence from our study supports such a statement, highlighting the difficult process of FMG identity creation. At the beginning, nothing changed in the group practice under study: physicians worked according to usual practices already in place and new material and human resources were only available at the end of the period. The FMG only existed in the minds of the group's champions, those who, very enthusiastically, saw in this reform project an opportunity to materialize their ideal vision of what better primary care delivery had to be for the sake of patients. This enthusiasm was so intense that, somehow, they forced the other physicians to embark on a project in which they were not very interested. Their actions (agency) led to a FMG without any sense of organizational self (structure).

During the second period, the pilot project inherited the label 'FMG' as well as the prevailing norms and values of individual practices and interprofessional distance between physicians and nurses. The difficulties of implementing the idea of FMG emerged very quickly. On one hand, physicians forced to adhere to the model kept practicing as usual, one of the champions left the unit, and the other one became progressively overwhelmed by his role. On the other hand, new FMG human resources (i.e. administrative and nursing staff) helped structure the central cornerstone of the FMG, physicians still acting on their own.

Power struggles between physicians and nurses characterized this second period. Values of interprofessional consensus and trust, and shared clinical practices toward the same clientele with respect to professional competencies appear to be supported only by the nurses; the physicians were too busy to stop and think about their practices, or found it difficult or uninteresting to do so. Furthermore, after a first unsuccessful step, the nurses adopted a position of retreat and resisted holding what was qualified a "religious" attitude vis-à-vis the physicians. Only between nurses and residents in family medicine did interprofessional clinical collaboration seem to emerge progressively without particular constraint.

The third FMG period came into being when a new director assumed leadership of the group. This was a female physician who had a rather practically oriented vision of the concept of a FMG. For her, the added value of a FMG was the presence of nurses collaborating with the physicians. Accordingly, she held not only legitimacy among her physician colleagues, but also among the cornerstone of the FMG, particularly the nurses. Hence, she tried to strongly support the emergence of new values of interprofessional dialogue and shared clinical practices, facilitating the joint presence of physicians and nurses in different internal forums as of April 2005 - i.e. regular FMG group meetings and case clinical discussions, and a one-day colloquium for discussing what their FMG "is and wants to be" scheduled in the fall of 2005. In other words, she instigated a process of *sensegiving*, which attempts "to influence the sensemaking and meaning construction of others towards a preferred redefinition of organizational reality" [34]. Thanks to the new leader's initiative, new spaces for *internal conversation *among the members of the FMG were created. Surrounded by a particularly disturbing institutional context, these new spaces were critical for this group of professionals to start shaping what they were becoming as well to be recognized as a distinctive group by other FMG stakeholders, patients included.

Our paper has a number of implications, for both research and practice. First, it demonstrates that the theoretical combination of structuration theory and critical discourse analysis - to our knowledge, never done before in studies on organizational identity - provides a rich picture of the process of identity construction. Second, our work makes a significant contribution to the sub-field of health services research in primary care when exploring the pervasive topic of teamworking from the perspective of team identity formation. Third, our work contributes to the still scarce but very promising health services research literature that challenges a traditional functionalist approach and emphasizes the important role to be played by sensemaking organizational processes for successful organizational innovation implementations [35] and health care reforms [36]. Fourth, the paper also contributes to qualitative research in family medicine, more particularly with regard to the use of discursive methodological approaches and techniques [37].

Indeed, a fundamental contribution of this study concerns practice. On the one hand, this investigation is the only one with an intense presence in the field of Quebec FGMs over a long period of time, and the only one that is examining the emergent identity of these groups of practice in the Quebec health care landscape. As such, through our research reports, this study has helped FMG practitioners better understand how they have initiated their FMG journey, what they are becoming, how they are constantly constructing this innovation, how they can learn from the consequences of their current practices, and, if appropriate, how to implement different actions intended to improve primary health care delivery in their particular context.

On the other hand, the results of this investigation are also helpful for decision-makers responsible for Quebec FMG implementation, as well as for those in charge of similar reforms in other jurisdictions. Regardless of policy interest in team-based organizational forms for primary health care delivery, building new care teams is a very fragile and challenging process. The present work highlights that initial policy definitions of who should integrate the team and what the team should do are not enough to support effective team development. In other to fulfil policy expectations, health professionals expected to work together need time and new spaces to communicate, interact and overcome interprofessional and interpersonal conflicts in the process of construction of *who they are as a team*, i.e. their family medicine group identity.

## Conclusions

In the present study, we have longitudinally assessed the emergence of an organizational innovation called family medicine group. We have particularly focused on the in-depth examination of the processes by which members of this team begin make sense to their new organizational reality. Regardless of the increased interest in team-based forms of organizing primary care delivery [31-33], the results of this investigation clearly highlights the difficulties of working together in interdisciplinary groups of practice. The creation of a primary care team cannot be taken for granted. On the contrary, it is a very challenging process that goes beyond the normative policy definitions of who is on the team or what the team has to do. To fulfil expectations in regard to quality improvement through team-based care, health care professionals meant to work together need shared time/space contexts to communicate, overcome interprofessional and interpersonal conflicts, and make sense and together define who they collectively are and what they do as a clinical team.

## Competing interests

The authors declare that they have no competing interests.

## Authors' contributions

CR conceived the study, organized the fieldwork, conducted the interviews, attended organizational and regional FMG meetings, collected documentary analysis, performed the analysis, and drafted the article. MP participated in the study as an expert in the methodological approach adopted, and contributed to the interpretation of results as well as in paper editing. Both authors read and approved the final manuscript.

## Pre-publication history

The pre-publication history for this paper can be accessed here:

http://www.biomedcentral.com/1471-2296/11/15/prepub
